# Success rates in smoking cessation: Psychological preparation plays a critical role and interacts with other factors such as psychoactive substances

**DOI:** 10.1371/journal.pone.0184800

**Published:** 2017-10-11

**Authors:** Bertrand Joly, Jean Perriot, Philippe d’Athis, Emmanuel Chazard, Georges Brousse, Catherine Quantin

**Affiliations:** 1 Biostatistics and Bioinformatics (DIM), University Hospital, Dijon, France; Bourgogne Franche-Comté University, Dijon, France; 2 Dispensaire Emile Roux, Centre d'Aide à I'Arrêt du Tabagisme (IRAAT), Centre de Lutte Anti-Tuberculeuse (CLAT), Clermont-Ferrand, France; 3 Univ. Lille, CHU Lille, Department of Public Health, Lille, France; 4 Psychiatry B-Department of Addictology, Université Clermont 1, UFR Médecine, Clermont-Ferrand, and CHU Clermont-Ferrand, Clermont-Ferrand, France; 5 INSERM, CIC 1432, Dijon, France; Dijon UniversityHospital, Clinical Investigation Center, Clinicalepidemiology/ Clinical trials unit, Dijon, France; 6 Biostatistics, Biomathematics, Pharmacoepidemiology and Infectious Diseases (B2PHI), INSERM, UVSQ, Institut Pasteur, Université Paris-Saclay, Paris, France; University of Montana, UNITED STATES

## Abstract

**Introduction:**

The aim of this study was to identify factors associated with the results of smoking cessation attempts.

**Methods:**

Data were collected in Clermont-Ferrand from a smoking cessation clinic between 1999 and 2009 (1,361 patients). Smoking cessation was considered a success when patients were abstinent 6 months after the beginning of cessation. Multivariate logistic regression was used to investigate the association between abstinence and different factors.

**Results:**

The significant factors were a history of depression (ORadjusted = 0.57, p = 0.003), state of depression at the initial consultation (ORa = 0.64, p = 0.005), other psychoactive substances (ORa = 0.52, p<0.0001), heart, lung and Ear-Nose-Throat diseases (ORa = 0.65, p = 0.005), age (ORa = 1.04, p<0.0001), the Richmond test (p<0.0001; when the patient’s motivation went from insufficient to moderate, the frequency of abstinence was twice as high) and the Prochaska algorithm (p<0.0001; when the patient went from the ‘pre-contemplation’ to the ‘contemplation’ level, the frequency of success was four times higher). A high score in the Richmond test had a greater impact on success with increasing age (significant interaction: p = 0.01). In exclusive smokers, the contemplation level in the Prochaska algorithm was enough to obtain a satisfactory abstinence rate (65.5%) whereas among consumers of other psychoactive substances, it was necessary to reach the preparation level in the Prochaska algorithm to achieve a success rate greater than 50% (significant interaction: p = 0.02).

**Conclusion:**

The psychological preparation of the smoker plays a critical role. The management of smoking cessation must be personalized, especially for consumers of other psychoactive substances and/or smokers with a history of depression.

## Introduction

Smoking is the leading cause of avoidable death in the world [1]. According to the World Health Organization, smoking kills 5 million people every year, that is to say more than HIV, tuberculosis and malaria together [2]. In France, smoking is the leading cause of premature death (before 65 years of age). The risk of anxiety and depression is also higher among smokers [3].

Despite these figures, smokers still find it difficult to stop smoking: 73% of smokers wish to stop; 22% try and less than 5% succeed without assistance [4]. Structured help with smoking cessation improves the chances of success of attempts to quit [5].

Many factors influence this success: the level of dependence, the number of cigarettes smoked, the consumption of other psychoactive substances (PAS), anxiety-depression disorders, weight gain and low motivation have a negative influence [6–10].

However, the following factors have a positive impact on abstinence: a late start in smoking, short duration of smoking or long periods without smoking (≥ 6 months), the perception of becoming a non-smoker within the following six months, a stable weight, high socio-economic and educational levels, a stable socio-professional status, a stable affective situation in a couple, no history of depression, and a non-smoker environment [6].

The aim here is to study the different factors that influence the results of cessation attempts so as to improve the management of such attempts. We conducted a multivariate analysis to identify, among factors related to success in univariate analysis, the strongest factors associated with abstinence. We compared our results with those in the literature so as to propose management adaptable to the specific characteristics of each patient.

## Materials and methods

According to the DIRECTIVE 2001/20/EC OF THE EUROPEAN PARLIAMENT AND OF THE COUNCIL of 4 April 2001 on the approximation of the laws, regulations and administrative provisions of the Member States relating to the implementation of good clinical practice in the conduct of clinical trials on medicinal products for human, when organizing a non-interventional non multicentre trial in France one does not need to ask for the authorization of an Ethics Committee but only to declare the study to the National Commission for Data Protection. This study was declared to the National Commission for Data Protection (CNIL n° 1873761) and our study adhered to the tenets of the Declaration of Helsinki.

We included all persons attending the Emile Roux dispensary in the”Pneumology and Tobaccology Centre” at Clermont-Ferrand, where assistance with smoking cessation was proposed between 1^st^ January 1999 and 31^st^ December 2009 (1,367 patients). These patients were referred to the centre by their primary care physician or encouraged to go there by their families. Smoking cessation could also be initiated by personnel of the centre during a visit to the dispensary for another health problem. The use of the information collected for this study was declared to the National Commission for Data Protection (CNIL n° 1873761) and our study adhered to the tenets of the Declaration of Helsinki.

In terms of management for each patient, there was an initial assessment either by a medical check-up with specific somatic, psychiatric or addictologic management, or by a nurse specialized in smoking cessation. At the end of the assessment, smoking management could be either immediate or deferred. In the case of delayed tobacco control, follow-up consisted of maintenance of motivation, possible treatment of anxiety-depressive disorders or of the consumption of other psychoactive substances, and possible reduction in consumption of tobacco with nicotine treatments. Each participant had access to cognitive-behavioral therapy, motivational interviews and pharmacotherapy (nicotine replacement therapy, or bupropion or varenicline, depending on the patient and on the period of follow-up of the study). In the case of exclusive pharmacological management based on nicotine substitutes, the transdermal sustained-release form was added to faster release oral gums, tablets or lozenges. This pharmacological treatment usually lasted 15 weeks. In addition to these systematic psychological and drug therapies, therapeutic education and treatment of anxiety-depressive disorders could be added depending on the clinical situation of the patient. During follow-up, each patient was also taken care of for withdrawal syndrome, the phenomenon of "craving" as well as the other side effects of the smoking cessation. Patient follow-up was reinforced with high availability of the team, including by telephone. Overall, follow-up was spread over 6 to 12 months before the final evaluation.

Regarding the population of our study, 1211 patients were treated with nicotine substitution, 177 with Bupropion and 79 with Varenicline (some patients had treatments with Bupropion and nicotine substitution or Varenicline and nicotine substitution), and 681 patients were also treated with an antidepressant with an average duration of 6.8 weeks. The mean duration of treatment with Bupropion and Varenicline was 3.1 weeks and 5.2 weeks, respectively. The mean duration of nicotine replacement therapy was 15.2 weeks with an initial mean dose of 26.6 mg / 24h.

The results of cessation were aggregated into two categories (failure and success), according to the patients’ declarations and the measurement of carbon monoxide level in exhaled gases 6 months after beginning. During the assisted smoking cessation, every patient filled in a personal follow-up notebook with his/her daily consumption of cigarettes, alcohol, coffee or other psychoactive substances. In France, smoking cessation clinics used the "tabacology file and notebook" proposed by the French Society of Tobaccology. Smoking cessation between two controls was checked at each follow-up visit by examining the notebook, and on the day of the visit by measuring expired CO. During the visit at 6 months, the patients were asked to provide information about their abstinence (self-reported in the notebook) and to perform expired CO testing (the CO test was done systematically). We concluded that the patient was abstinent if the CO level was less than 7 ppm and if abstinence over the past month was reported in the notebook. Smoking one puff per day was counted as failure, as was the case if the notebook was not presented or if expired CO level was above the threshold values.

The studied factors were age, sex, daily consumption of cigarettes (when smoking cessation began), number of cigarette-pack years, consumption of another psychoactive substance, number of previous cessation attempts, duration of the longest temporary abstinence, a history of major depression, current state of depression at baseline, current state of anxiety at baseline; smoking-related heart, lung or Ear-Nose-Throat (ENT) diseases; the Fagerström score (nicotine addiction test) [11], the total score of the Richmond test (an indicator of motivation to quit smoking, motivation was considered ‘insufficient’ when the score was from 0 to 5, ‘moderate’ when the score was from 6 to 8 and ‘high’ when the score was 9 or 10),) [12,13] (Appendix A in S1 Appendix), level according to the Prochaska and Di Clemente algorithm (indicator of success in smoking cessation attempt with five levels [14]: ‘Pre-contemplation’, ‘Contemplation’, ‘Preparation’, ‘Action’ and ‘Maintenance’. Six patients had reached the ‘maintenance’ stage at the initial consultation: they may have begun smoking cessation before this consultation and, as a consequence, were excluded. All of the studied factors were measured at the initial consultation. Regarding the state of anxiety, only a current state of anxiety was measured at baseline. States of current anxiety and current depression at baseline were measured with ‘the hospital anxiety and depression scale’ [15,16] (Appendix B in S1 Appendix). Regarding depression, both a current state of depression at baseline and a history of depression were measured. The history of depression was defined by a treatment for depression for a period of more than three months and self-reported by the patient during the visit at baseline (patients were asked to provide more information about their treatment and the follow-up for this previous episode of depression). We considered a single variable ‘depression’, recoded with these three modalities (history of depression, current state of depression, never depression). We coded as a “current state of depression” any patient with both a history of depression and a current state of depression at baseline.

Between-group comparisons of distributions for categorical data (Richmond tests, Prochaska and DiClemente) were done with the Chi^2^ test. Student’s t test was used to compare means (number of cigarettes; Fagerström). Multivariate logistic regression was used to investigate the association between abstinence and other factors. To check the absence of collinearity, we computed correlation coefficients for all pairs of variables. All variables were included in one step. The variable « duration of the longest temporary abstinence » was excluded due to the absence of convergence of the iterative process of estimation. Interactions between significant variables in the regression analysis (p<0.05) were searched for two by two. The area under the ROC curve (AUC) and the Hosmer-Lemeshow goodness-of-fit test were calculated for the final model. The data were analysed using R 2.10.1 software.

### Human ethics

This was a non-interventional study as all of the procedures and all of the products were those used in routine care. No additional or unusual diagnostic or surveillance procedures were carried out. The assignment of a patient to a given medical strategy was not established in advance by a protocol and was simply routine practice. Patients came to the Emile Roux dispensary for a usual consultation.

In France, consent for non-interventional studies is not compulsory, but a single-centre non-interventional study requires authorization from the data protection agency (CNIL).

The use of the information collected for this study was then declared to the National Commission for Data Protection (CNIL n° 1873761). The nominative data relative to care were collected by the doctors at the Emile Roux dispensary as declared to the CNIL. Before conducting the statistical processing necessary for the study, the data were anonymized.

## Results

### Univariate and bivariate analyses

#### Failure or success of the cessation attempt as a binary variable

Of the 1,361 patients, 630 failed in their cessation attempt and 731 succeeded. Sex was not significantly related to abstinence. A current state of anxiety at baseline (p<0.0001) was significantly associated with a decreased frequency of abstinence. The consumption of a psychoactive substance other than tobacco (p<0.0001) and the presence of a heart, lung or ENT disease (p<0.0001) also diminished the frequency abstinence. In contrast, a previous attempt to stop smoking increased the frequency of success (p = 0.001). (Table 1)

**Table 1:** Frequencies (at the initial consultation) according to abstinence.

	Failure (n = 630)	Success (n = 737)	*p value*	Chi_square
Sex (male)	50.2%	46.4%	0.15	2.1
Current state of anxiety at baseline	46.8%	29.3%	<0.0001	45.2
Previous attempt	76.5%	83.4%	0.0008	11.2
Other psychoactive substances	40%	18.7%	<0.0001	73.8
Heart, lung and Ear-Nose-Throatdiseases	57.8%	44.2%	<0.0001	25

The variable depression was significantly associated with abstinence (p<0.0001 with Chi-square = 68.9). A history of depression (p = 0.0002 with Chi-square = 14.1) and current state of depression at baseline (p<0.0001 with Chi-square = 67.2) were all significantly associated with a decreased frequency of abstinence compared to never depression.

The higher the level of the Richmond test, the greater the chances of success (p<0.0001 with Chi-square = 151.1): 68.7%of smokers with ‘high’ motivation and 59.6% of smokers with ‘moderate’ motivation at the Richmond test succeeded in their attempt compared with 24.2% among smokers with ‘insufficient’ motivation.

The more advanced the patient in the Prochaska algorithm, the greater the chances of success in stopping smoking (p<0.0001 with Chi-square = 146.4): 57.6% of smokers at the ‘contemplation’ level succeeded in their cessation attempt compared with 12.4% of those at the ‘pre-contemplation’ level.

The number of cigarettes smoked every day was significantly associated with the abstinence rate (p<0.001), which diminished with increasing numbers of cigarettes smoked. The age (p = 0.0002), the Fagerström index (p<0.0001), the duration of the longest abstinence (p<0.0001)had a significant positive impact on abstinence. Neither the number of previous attempts to stop smoking nor the number of pack-years (which depends on both age the daily consumption of cigarettes) was a significant factor. (Table 2)

**Table 2:** Association between abstinence and each quantitative^1^ variable at the initial consultation.

		Failure	Success		T-test value
Variables	n	Mean (SD)	Mean (SD)	*p value*	
Age	1,338	43.6 (11.3)	45.9 (11.4)	0.0002	3.7
Fagerström Index	1,359	7.7 (1.9)	7 (2.1)	<0.0001	6.7
Number of cigarettes per day	1,358	26.2 (11.5)	23.5 (10.2)	<0.0001	4.6
Number of pack-years	1,354	30.4 (18.9)	28.9 (18.3)	0.13	1.5
Number of prior attempts to quit	1,321	2.2 (3)	2.3 (2.5)	0.36	0.9
Duration of longest temporary abstinence	1,078	7.1 (14.7)	11.6 (19.1)	<0.0001	4.4

1: the results corresponding to qualitative variables are given in the text

### Multivariate analysis

We found a significant association between current state of anxiety at baseline and a state of depression. However, as these variables do not represent the same information from a clinical point of view, we decided to take them into account separately in the multivariate analysis.

As there were 29 individuals with missing data, 1,332 individuals were included in this multivariate analysis.

We computed correlation coefficients for all pairs of variables, and all were lower than 0.61.

Depression remained a significant obstacle to abstinence (p = 0.004). In contrast, current state of anxiety at baseline, a previous attempt at smoking cessation, the Fagerstrom score and the number of cigarettes smoked every day were no longer significant in multivariate analysis. (Table 3)

**Table 3:** Variables (at the initial consultation) included in the multivariate analysis (significant in univariate analysis), with abstinence (binary form 1 if success, 0 if failure).

	ORa	95% CI	P
**Depression**			**0.004**
Current **state of depression at baseline**	0.64	**0.47–0.88**	0.005
History of depression	0.57	**0.39–0.83**	0.003
**Current state of anxiety at baseline**	0.77	**0.58–1.02**	0.09
**Previous attempt to quit**	0.97	**0.70–1.35**	0.97
**Other psychoactive substance**	0.52	**0.39–0.70**	<0.0001
**Heart, lung or Ear-Nose-Throat disease**	0.65	**0.49–0.86**	**0.005**
**Age**	1.04	**1.03–1.06**	<0.0001
**Fagerstr**ö**m Index**	0.95	**0.88–1.03**	0.22
**Number of cigarettes per day**	0.99	**0.98–1.01**	0.48
**Richmond Test**			<0.0001
**Passage from insufficient motivation to moderate motivation in the Richmond test**	2.18	**1.50–3.16**	**<0.0001**
**Passage from moderate motivation to high motivation in the Richmond test**	1.26	**0.88–1.80**	**0.21**
**Prochaska algorithm**			<0.0001
**Passage from the " pre-contemplation" level to the " contemplation" level**	4.53	**2.59–7.91**	<0.0001
**Passage from the "contemplation" level to the "preparation" level**	0.89	**0.60–1.31**	**0.55**
**Passage from the "preparation" level to the "action " level**	2.18	**1.23–3.87**	0.008

An older age was a highly significant positive factor for abstinence (p<0.0001). The total score in the Richmond test (p<0.0001) and the level in the Prochaska algorithm (p<0.0001) remained highly significant, each of which had a positive impact on abstinence.

The consumption of another psychoactive substance (p<0.0001) or the presence of heart, lung or ENT disease (p = 0.005) remained highly significant barriers to abstinence.

Two significant interactions were found by bivariate analyses:

- between the score in the Richmond test and age (p = 0.01): the difference between low motivation and moderate motivation was less marked in smokers aged 45 years or less (30%) than in smokers more than 45 years old (40%). An increase in the score in the Richmond test therefore had a greater impact in older smokers. (Table 4)

**Table 4:** Frequency of abstinence according to age and the Richmond test at the initial consultation.

	Richmond ‘insufficient’ motivation	Richmond ‘moderate’ motivation	Richmond ‘high’ motivation
**Age less than or equal to the median (45 years)**	26.1%	55.5%	59.3%
**Age above the median (45 years)**	23.5%	64.1%	78.6%

- between the consumption of other psychoactive substances and the level in the Prochaska algorithm (p = 0.02): when smokers had reached at least the ‘preparation’ level in the Prochaska algorithm, the chances of abstinence were reduced when patients consumed another psychoactive substance. Among exclusive smokers, the contemplation level was sufficient to obtain a satisfactory abstinence rate (65.5%) whereas smokers who consumed other psychoactive substances had to reach the preparation level to achieve a success rate greater than 50%. (Table 5)

**Table 5:** Frequency of abstinence according to consumption of another psychoactive substance and the level in the Prochaska algorithm at the initial consultation.

	Prochaska ‘re-contemplation”	Prochaska ‘contemplation’	Prochaska ‘preparation’	Prochaska ‘action ‘
**No other psychoactive substance consumed**	19.1%	65.2%	60.6%	77.2%
**Another psychoactive substance consumed**	4.9%	37.7%	58.3%	72.7%

These two interactions, when included together in the model, remained significant (p = 0.03 for the first and p = 0.04 for the second).

Concerning the Prochaska algorithm, the Richmond test, we examined AUC (Area Under the ROC Curve) changes resulting from adding any one or twoof these variables to the model. AUCs were very similar and varied from 0.71 (without the variables: Prochaska algorithm, Richmond test) to 0.76 (with the 2 variables: Prochaska algorithm, Richmond test) and 0.78 (with the variables: Prochaska algorithm, Richmond testand the two interactions detected). The Hosmer-Lemeshow goodness-of-fit test of the final model with the two interactions was in favor of good calibration (p = 0.77).

## Discussion

Women and men did not differ significantly in tobacco abstinence outcomes [17]. However, an older age was a highly significant positive factor of abstinence; this has already been reported in the international literature [18,19]. What can explain this result? Perhaps the burden of smoking is perceived with more realism, or it could be that the illusions peddled by tobacco consumption are seen for what they are with time, and the first obvious adverse effects of smoking are felt. It is also possible that the fluctuations surrounding any decision and any motivation could perhaps diminish with age.

In our study, as in the international literature [20–24], psychiatric symptoms were related to relapse. Other studies have shown that adding mood management to behavioural support may improve abstinence in smokers with current or past depression [25–27]. But one interesting aspect of our study was that ‘a history of depression’ had a greater impact than did ‘current state of depression. This could be the result of the management of ‘current state of depression at baseline’ during the study, either by the use of antidepressants or other treatments, which was not necessarily the case for ‘a history of depression’. Unlike these two variables, 'current state of anxiety at baseline' was no longer significant in multivariate analysis. This may have been due to proper management of anxiety state during the study or to a lack of statistical power. The above suggests that a history of depression reflects persistent psychological vulnerability that may foster a return to smoking. The fact that a history of depression remained significant in multivariate analysis agrees with the international literature, and shows that current state of depression or current state of anxiety at baseline are not, in terms of psychological dysfunction, the strongest factors associated with a failed smoking cessation attempt [28,29]. In addition, it has been shown that a deteriorated psychological status after smoking cessation does not increase the risk of a relapse to smoking [30]. Other studies are needed to confirm that a history of depression is a more precise and more reliable factor associated with a relapse to smoking than are current state of anxiety and current state of depression at baseline.

Our study showed that a smoking-related disease did not facilitate smoking cessation or the maintenance of abstinence [31–33]. Yet the literature is divergent on this matter. Azevedo, for example, reported the opposite: a lack of success correlated with the absence of a smoking-related disease [34]. The diseases listed in the Azevedo study, however, (any type of smoking-related cancer) were not the same as ours. The principal studies showing that disease has a positive impact on smoking cessation principally concern patients undergoing treatment for cancer [35–38]. The diseases considered in our study were all smoking-related heart, lung and ENT diseases. The question arises as to whether these patients were discouraged from stopping as the disease was already there. Because of this phenomenon, the efficacy of cognitive-behavioural therapies may have been diminished. Moreover Van Eerd showed that smokers with COPD (Chronic obstructive pulmonary disease) could be indignant about a perceived lack of empathy from doctors [39]. In these specific patients, it could be interesting to insist on the benefits of stopping smoking with regard to these diseases and to measure the impact of this insistence.

As for the Prochaska algorithm, moving from the pre-contemplation level to the contemplation level was associated with a greater frequency of stopping smoking: this change in level should thus be pursued before starting the cessation process. In addition to the motivation interview, the use of pre-cessation Nicotine replacement therapy (NRT) or another intervention that increases motivation, self-efficacy and self-esteem could facilitate this level change [40–42]. Concerning the Richmond test, the passage from ‘low’ to ‘moderate’ motivation gives the greatest improvement in the chances of abstinence. As ‘moderate’ motivation is necessary to obtain a satisfactory chance of success, this level of motivation must be pursued before starting the cessation process [43,44]. Increasing from ‘moderate’ to ‘high’ motivation on the Richmond test did not seem to have a significant impact on abstinence. This seems to show that the passage from ‘insufficient’ to ‘moderate’ motivation on the Richmond test is key factor to obtain frequent abstinence in clinical practice. The level in the Prochaska algorithm and the total score of the Richmond testwere both highly significant factors (both p values were less than 0.0001). These two notions reflect the psychological preparation of patients vis-a-vis quitting smoking, which underlines the importance of psychological preparation before beginning the cessation.

It must be pointed out, however, that these two tests were simultaneously significant in the same model, indicating that they clearly measure two different notions: motivation for the Richmond test and maturation of the decision for the Prochaska algorithm. Other studies are necessary to determine whether the evolution of the scores for these two different tests during the cessation process could predict a relapse to smoking or abstinence.

The overwhelming importance of this psychological preparation in abstinence underlines the interest of cognitive-behavioural therapies as a support in attempts to stop smoking [45,46]. These therapies can also be associated with the use of NRT to facilitate the reduction in consumption before abstinence [47]. Moreover, other studies have shown that other psychological components interfere with the result of smoking cessation attempts. In fact, motivation to attend behavioural support sessions is distinct from motivation to quit smoking [48]. And such interventions to increase adherence to medication for tobacco dependence seem to improve the chances of achieving abstinence [49]. In the same way, another study suggested that repeated counselling about goals is advisable and smokers would benefit from such counselling [17]. Maintaining motivation at a sufficiently high level therefore appears to be a key element in abstinence. In the context of smoking cessation, not all of the psychological components on which behavioural support must focus have been identified, but each will require additional investigations. Behavioural support is still an avenue of research to improve abstinence [50–52].

To our knowledge, this study is the first to report the interaction between the score in the Richmond test and age: the greater the age, the greater the positive impact of the Richmond score on abstinence.

The consumption of another psychoactive substance diminishes the chances of abstinence [28,53–56]. It is possible that during co-addiction, the transfer of one addiction to another explains this decrease. Moreover, this study is also the first to report the interaction between the level in the Prochaska algorithm and the consumption of another psychoactive substance. In our study, abstinence was lower in smokers who also consumed another psychoactive substance, but the difference decreased with progress in the Prochaska algorithm. Thus, for tobacco only smokers, the ‘contemplation’ level of the Prochaska algorithm was enough to obtain a satisfactory success frequency (65.2%). For consumers of another psychoactive substance, smokers had to reach the ‘preparation’ level of the Prochaska algorithm to obtain a success frequency greater than 50%. This relationship was very strong and the interaction with the level in the Prochaska algorithm suggests that smokers who consume another psychoactive substance deserve particular attention from the support provider. A reinforced motivational interview concerning smoking and the associated psychoactive substance could be a way to improve management. It is thus essential to set up pre-cessation strategies to ensure that consumers of other psychoactive substances reach this ‘preparation’ level of the Prochaska algorithm at the start of the cessation attempt. The question arises as to whether the fluctuations in decision-making and motivation could be greater in these patients than in exclusive tobacco smokers. Another study showed the existence of an interaction between treatment with antidepressants and the consumption of another psychoactive substance during smoking cessation [54]: taking an antidepressant had a positive impact on abstinence in smokers who consumed another psychoactive substance but not in exclusive smokers. Taking another psychoactive substance thus appears to disturb the mechanisms of classical smoking cessation strategies.

### Strengths and limitations of our study

One limitation of our study is that the CO cut-off should have been < 5 ppm and that the level of 7 pm is now considered too liberal to ensure no smoking. However, this study began in 1999 and was finished in 2009, when the use of the level 7 ppm was still the rule. Another limitation of our study is that we only investigated factors already reported in the international literature, which were for the most part either instruments to measure the psychological preparation of patients for abstinence or factors principally seen as barriers to abstinence. At no time in our study did we include positive factors or incentives to stop smoking; this is also the case in most international studies. These potential incentives could also have an impact on the result of the cessation attempt: the fact of finding a job, starting to live in a couple, getting a promotion or any other type of positive event in life have not been studied and could prove to be propitious moments to embark on and succeed in smoking cessation. The strength of our study is first of all that the significant results as yet unreported may be combined with results in the international literature. The simultaneous and independent effects of two tests that measure the psychological preparation of the patient bring to light the importance of this mental preparation before starting the cessation attempt, while the interactions of these tests with age on the one hand, and the consumption of another psychoactive substance on the other, show the need to adapt this preparation to the context. These results were obtained in a large number of patients, thus conferring sufficient statistical power. The long duration of our study is also a strong point because it incorporated changes in medical practice, which is not the case in short studies, either single-blinded or experimental. Our observational study thus assessed the management of smokers in a real-life setting in smoking cessation centres.

## Conclusion

This study showed that motivation and maturation of the patient in the decision-making process play an essential role in the success of the cessation attempt. It is crucial to help patients to reach a sufficient level of motivation (at least moderate) and to achieve optimal maturation of the decision-making process at the start of the attempt to stop smoking, and to maintain this state throughout the abstinence.

This study also showed the existence of interactions and that the support provided to help smokers stop smoking must be adapted to the profile of the smoker and take into account the existence of a current state of depression at baseline, a history of depression, the presence of a smoking-related somatic disease and the use of other psychoactive substances. Smokers who consume another psychoactive substance seem to need, above all, appropriate preparation to allow them to reach a higher Prochaska level than that needed in exclusive smokers, as well as personalized management and reinforced follow-up.

## Supporting information

S1 Appendix Appendix A: Richmond test. Appendix B: The hospital anxiety and depression scale.
(DOCX)
