# Supplementary material for: Success rates in smoking cessation: Psychological preparation plays a critical role and interacts with other factors such as psychoactive substances
Source: PLoS One. 2017 Oct 11;12(10):e0184800. doi: 10.1371/journal.pone.0184800 (PMC5636087; doi:10.1371/journal.pone.0184800)
Supplement: S1 Appendix — Appendix A: Richmond test. Appendix B: The hospital anxiety and depression scale. (DOCX) [file pone.0184800.s001.docx]

**Appendix A: Richmond test**

Motivation Assessment Test

RL RICHMOND ADDICTION, 1993, 88 : 1127-35 (12)

| Would you like to quit smoking? | no  yes | 0  1 |
| --- | --- | --- |
| Do you really want to quit smoking? | not at all  a little  moderately  a lot | 0  1  2  3 |
| Do you think you will quit smoking  in the next 4 weeks? | no  perhaps  probably  certainly | 0  1  2  3 |
| Do you think you will be an ex-smoker within 6 months | no  perhaps  probably  certainly | 0  1  2  3 |

**0- 5 ‘**insufficient’ motivation  **6- 8** ‘moderate’ motivation 9-**10** ‘high’ motivation

**Appendix B:** **[The hospital anxiety and depression scale](https://www.ncbi.nlm.nih.gov/pubmed/3080166)**[.](https://www.ncbi.nlm.nih.gov/pubmed/3080166)

SELF-EVALUATION SCALE HAD

Please circle the scores that apply to you

1. I feel tense, edgy

Most of the time …………………………………………………………………………………. 3

Often ……………………………………………………………………………………………….. 2

From time to time ………………………………………………………………………………….. 1

Never ………………………………………………………………………………………………… 0

1. I always have as much fun doing things that I like

Yes, always ………………………………………………………………………………………….. 0

Most of the time ………………………………………………………………………………………. 1

More and more rarely …………………………………………………………………………… 2

Everything is more difficult ……………………………………………………………………………….… 3

1. I feel slowed down

Practically all the time …………………………………………………………………………... 3

Very often …………………………………………………………………………………………… 2

Sometimes ……………………………………………………………………………………………. 1

Not at all …………………………………………………………………………………………….. 0

1. I feel worried and have a lump in my throat or a knot in my stomach

Very often.………………………………………………………………………………………….. 3

Quite often …………………………………………………………………………………………. 2

Sometimes ………………………………………………………………………………………………….. 1

Never ………………………………………………………………………………………………….. 0

1. I’ve lost interest in my appearance

Totally ……………………………………………………………………………………………… 3

I'm not paying attention to it ………………………………………………………………………………. 2

I do not pay enough attention …………………………………………………………………….…. 1

I take care of it as usual ………………………………………………………………….. 0

1. I feel restless and can’t keep still

Yes, that’s exactly right ………………………………………………………………...………….. 3

A little …………………………………………………………………………………………………… 2

Not so much …………………………………………………………………………………………… 1

Not at all ……………………………………………………………………………………………… 0

1. I am look to the future with optimism

As usual …………………………………………………………………………………….. 0

Rather less than before …………………………………………………………………………….……. 1

Much less than before …………………………………………………………………………….. 2

Not at all ……………………………………………………………………………………………… 3

1. I feel afraid, as if something terrible is going to happen to me

Yes, absolutely ……………………………………………………………………………………. 3

Yes, but it is not too serious ……………………………………………………………………. 2

A little, but it doesn’t bother me ………………………………………………………………… 1

Not at all …………………………………………………………………………………………….. 0

1. I can laugh and see the good side of things

As much as usual……………………………………………………………………………………….. 0

Rather less ………………………………………………………………………………………….… 1

Much less ……………………………………………………………………………………… 2

Not at all ……………………………………………………………………………………………. 3

1. I often worry

Very often ……………………………………………….…………………………………………. 3

Quite often ………………………………………………………………………………………… 2

Occasionally …………………………………………………………………………………… 1

Very occasionally ……………………………………………………………………………… 0

1. I feel happy

Never ………………………………………………………………………………………………… 3

Not often ………………………………………………………………………………………….. 2

Sometimes …………………………………………………………………………………………… 1

Most of the time …………………………………………………………………………………. 0

1. I can sit down and do nothing and feel happy

Never ………………………………………………………………………………………………… 3

Rarely …………………………………………………………………………………………….. 2

Yes, in general ………………………………………………………………………………………. 1

Yes, whatever happens …………………………………………………………………………………. 0

1. I enjoy a good book or a good radio or TV program

Often ………………………………………………………………………………………………. 0

Quite Often ……………………………………………………………………………………….. 1

Rarely ……………………………………………………………………………………………... 2

Hardly ever ………………………………………………………………………………… 3

1. I have sudden panic attacks

Very often …………………………………………………………………………………………. 3

Quite often ……………………………………………………………………………………….. 2

Rarely ……………………………………………………………………………………………... 1

Never ………………………………………………………………………………………………… 0

# EVALUATION GRID

| **ANXIETY** | | **DEPRESSION** | |
| --- | --- | --- | --- |
| **QUESTION N°** | **SCORE** | **QUESTION N°** | **SCORE** |
| **1** |  | **2** |  |
| **4** |  | **3** |  |
| **6** |  | **5** |  |
| **8** |  | **7** |  |
| **10** |  | **9** |  |
| **12** |  | **11** |  |
| **14** |  | **13** |  |
| **TOTAL ANXIETY :** | | **TOTAL DEPRESSION :** | |

**To diagnose anxiety, via dimension A of ‘the hospital anxiety and depression scale’, the sub-score had to be greater than 7. To diagnose depression, via dimension D of ‘the hospital anxiety and depression scale’, the sub-score had to be greater than 11. It was also necessary to combine the clinical criteria of the DSM IV in addition to the appropriate scores on the ‘the hospital anxiety and depression scale’**
